# Assessment of suicidal behavior and factors associated with a diagnosis of prostate cancer

**DOI:** 10.6061/clinics/2018/e441

**Published:** 2018-11-06

**Authors:** Marilia Zendron, Stênio C Zequi, Gustavo C Guimarães, Maria Teresa C Lourenço

**Affiliations:** I Programa de Pos Graduacao Fundacao Antonio Prudente, A.C. Camargo Cancer Center, Sao Paulo, SP, BR; II Divisao de Urologia, A.C. Camargo Cancer Center, Sao Paulo, SP, BR; III Departamento de Psico-Oncologia, A.C. Camargo Cancer Center, Sao Paulo, SP, BR

**Keywords:** Anxiety, Depression, Diagnosis, Prostate Cancer, Suicide

## Abstract

**OBJECTIVES:**

To determine the incidence of suicide risk in a group of patients who have been diagnosed with localized prostate cancer (PC) and to identify the factors that affect suicidal behavior.

**METHODS:**

Patients from a tertiary care oncology center in São Paulo, Brazil participated in this study and were interviewed after being diagnosed with low-risk or intermediate-risk PC, per the D'Amico risk classification, between September 2015 and March 2016. Patients underwent suicide risk assessment sessions using the Mini International Neuropsychiatric Interview (MINI), the Hospital Anxiety and Depression Scale (HADS), and the CAGE substance abuse screening tool before they started treatment and surveillance. Psychiatric treatment history, family history of suicidal behavior, and the use of psychotropic drugs were also examined.

**RESULTS:**

The prevalence of suicide risk among 250 patients who were recently diagnosed with low-risk or intermediate-risk PC was 4.8%. According to the HADS, 10.8% and 6.8% of patients had a positive score anxiety and for depression, respectively. Alcoholism was suspected in 2.8% of the group. Suicide risk was associated with anxiety (*p*=0.001); depression (*p*=0.005); being divorced, separated, widowed, or single (*p*=0.045); living alone (*p*=0.028); and prior psychological treatment (*p*=0.003).

**CONCLUSIONS:**

After being diagnosed with PC, patients who display risk factors for suicide should be monitored by a mental health team.

## INTRODUCTION

In the past several decades, there has been an increase in the number of cancer diagnoses worldwide. Data from the WHO predict that 27 million people will develop cancer by 2030. In Brazil, the National Cancer Institute (INCA) estimates that 600,000 new cases will have developed in 2016-2017, with 61,200 new cases of prostate cancer (PC) and 13,772 deaths from PC 1. In the US, 161,360 new PC cases and 26,730 deaths from PC are expected to occur annually 2.

The most common treatments for PC are surgery, radiotherapy, and hormone therapy. These modalities affect sexual 3,4, urinary 5, and intestinal function 4 to varying degrees, which often compromises the quality of life of patients and impacts their mental health 5. Low-risk cases might be candidates for medical surveillance, requiring the patient to undergo periodic medical visits, evaluations of prostate-specific antigen (PSA) levels, rectal examination, magnetic resonance tests, and repeated transrectal prostate biopsies 6.

Cancer patients can be at significant risk of suicide—2-fold higher than the risk in the general population 7-9. Suicide rates are greater during the first several months after the diagnosis, peaking in the first month following discovery of the illness 8. Suicidal behavior encompasses suicide gestures, suicide attempts, and successful suicides. Suicide gestures have little chance of leading to death, whereas attempts imply an action with the intent to cause death, although this objective is not met; in contrast successful suicides result in death 10.

A Brazilian study demonstrated that 1 in 5 oncologic patients experiences depression and that 5% of these subjects are at risk for suicide in association with pain or depression 11. Few studies have examined the risk of suicide in cancer patients, specifically those with PC. The suicide rate is high (6.5%) in these patients during the first 6 months after diagnosis, independent of the treatment method 12. Even after treatment, the prevention and management of anxiety, depression, and suicidal behavior remain critical, because patients who undergo radiation or chemotherapy have a higher prevalence of depression, and age can influence psychological stress 9.

The purpose of this study was to determine the incidence of suicidal risk and identify the risk factors for suicidal behavior in a group of patients who have been recently diagnosed with PC.

## MATERIALS AND METHODS

### Study design

This prospective study was performed in patients from the Urology Center at A.C. Camargo Cancer Center, including private patients, subjects in a supplementary health system (insured patients), and uninsured patients who are supported by the Brazilian Public Health System (Sistema Único de Saúde – SUS). The project was approved by the institution's ethics committee under protocol number 1.169.723.

### Participants

Two hundred sixty-four patients were asked to participate in this study between September 2015 and November 2016 after being diagnosed (between 1 and 6 months after diagnosis) with low-risk or intermediate-risk PC, per the D'Amico risk classification 13.

### Analytical tools

We gathered the following sociodemographic data: age, race, religion, marital status, education level, cohabitation status, personal and family history of cancer, smoking status at the time of the interview, alcohol consumption, recommended treatment (surgery, radiation therapy, high-intensity focused ultrasound - HIFU, or active surveillance), personal psychiatric history, family history of suicidal behavior (including suicide attempts and completions), and psychiatric drug use. Regarding religious practices, patients who described themselves as not ascribing to any religion were considered atheists. The other patients were placed in the “religious” category. Three questionnaires were given before the patients started treatment: the Hospital Anxiety and Depression Scale (HADS) 14,15, the CAGE questionnaire (used to determine alcohol abuse and dependence) 16,17 and The Mini International Neuropsychiatric Interview (MINI) 18,19.

Patients who presented with a risk of suicide were referred to the psycho-oncology center for evaluation and psychiatric treatment.

### Statistical analysis

The quantitative variables were measures of central tendency and dispersion, and the qualitative variables were absolute and relative frequencies (percentages). The association between the 2 qualitative variables was analyzed by Fisher's exact test, and the nonparametric Mann-Whitney U test was performed to compare the 2 groups of quantitative variables.

An adjusted logistic regression model was used to evaluate the factors that influenced the presence or absence of suicide risk (chance), based on their odds ratios and confidence intervals (95% CIs). In the multivariate analysis, variables were considered to be covariates when the association test yielded a *p*-value <0.05. Statistics analysis were performed using the Statistical Package for Social Sciences (SPSS), version 22.0.

## RESULTS

Six patients refused to participate; 7 were excluded because they were reclassified as being high-risk after a review of their tissue sections, and 1 was excluded for presenting with cognitive impairments. Patients had an average age of 62.6 years (range: 38 to 88 years). The average time between the diagnosis and interview was 68 days (standard deviation: 40.03 days). The incidence of suicide risk for patients who were recently diagnosed with PC was 4.8%. Based on the subdivisions per the cut-offs for the MINI questionnaire, the incidence of suicide risk was 3.2% for low-risk suicide cases and 0.8% for moderate- and high-risk suicide cases.

Between the HADS, CAGE, and MINI questionnaires, only the HADS results confirmed a correlation with suicide risk (*p*=0.001 for anxiety and *p*=0.005 for depression; Table 1). Regarding the link between suicide risk and sociodemographic data, we noted a relationship with marital status (*p*=0.045), living alone (*p*=0.028), and prior psychiatric treatment (*p*=0.003). No other variable was associated with suicide risk (Table 2).

An adjusted logistic regression model was used to assess suicide risk (yes and no). The covariates for the univariate analysis were as follows: living alone, prior psychiatric treatment, HADS with a positive score for anxiety (HADSa - yes and no), and HADS with a positive score for depression (HADSd - yes and no). Although marital status was correlated significantly with suicide risk (*p*=0.045), we did not include this variable in our model, because we believe that these data could be represented by the variable “living alone”—93.3% of patients who lived alone were divorced, separated, widowed, or single, and 86.8% of patients who were not living alone were married. Using this model, we estimated the probability of suicide risk (Table 3) as follows: 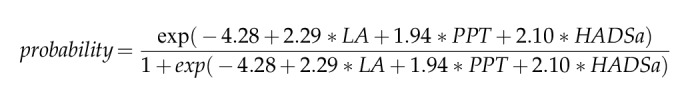


where LA=1 if the patient lived alone and LA=0 if he did not; PPT=1 if the patient underwent prior psychiatric treatment and PPT=0 if he did not; and HADSa=1 for a positive score for anxiety and HADSa=0 for a negative score (Table 3).

## DISCUSSION

Our study evaluated suicide risk in a specific oncological population using an expressive approach with well-defined criteria: patients who had been diagnosed within 6 months with low-risk or intermediate-risk PC and who had not initiated treatment.

The factors that we found to be associated with suicide risk in our sample are consistent with those in the literature: marital status 12,20-23, living alone 5, prior psychiatric treatment 9,22, and a positive HADS score 9. The prevalence of suicide risk among our patients was 4.8%, similar to what was reported by Fanger et al. 10. However, this group interviewed patients who had been admitted for various types of cancer and did not specify the stage of the illness.

Patients with PC were classified as having a high level of pretreatment anxiety 24,25. Our survey revealed that 22.2% of patients with anxiety and 23.5% of patients with depression exhibited suicide risk. Depressive disorder and anxiety are not always diagnosed. Thus, questionnaires can identify feelings that are not clearly expressed by the patient. Many patients experience a period of adjustment to their new situation and should therefore be monitored more closely by the management team.

A total of 20.8% of our patients with previous psychiatric disorders were at risk for suicide (*p*=0.003). They were taking medications to treat anxiety, depression, stress, panic disorder, and burnout syndrome.

Similar to other studies, we found that single, divorced, or widowed patients were at a higher risk of suicide (11.1%) after being diagnosed with cancer than were married patients (3.4%) 23. Further, among patients who lived alone, 20% were at risk for suicide (*p*=0.028). Lehuluante and Fransson 5 reported a significant relationship between not being married or living without a partner and suicidal thoughts in prostate cancer patients. Erlangsen et al. 26 noted that men with any physical illness who were married or lived with someone had a suicide rate that was below that of men who were single, divorced, or widowed. These findings suggest that not living alone helps a patient endure treatment for PC.

The interviews and the use of questionnaires before the cancer treatment, as described, could allow us to detect the patients at risk of suicide and provide better psychological support for these patients during the entire disease trajectory.

Study implications: We believe that assessments should be performed to evaluate suicide risk in 3 phases: after diagnosis, immediately after treatment, and in the subsequent period (surveillance). The HADS could serve as the preferred instrument to initially evaluate this population.

Study limitations: Probable clinical comorbidities and illicit drug use were not examined in this study, despite their influence on suicidal behavior 23,27. Thus, these factor merit consideration in future research.

No Brazilian study has assessed the risk of suicide among patients who have recently been diagnosed with low-risk PC. Our study underscores the need for special care from the clinical team to identify patients who are at risk for suicide at the time of the diagnosis of a malignant neoplasm of the prostate and to refer them for a psychological and psychiatric evaluation.

## AUTHOR CONTRIBUTIONS

Zendron M was the main researcher, responsible for the data collection, manuscript writing, table elaboration and submission procedures of the manuscript. Lourenço MT supervised and provided instructions during the study and was responsible for the manuscript revision, data analysis and interpretation of data. Zequi SC was responsible for the manuscript revision and table elaboration. Guimarães GC approved the final version of the manuscript to be published. All authors have read and approved the final version of the manuscript.

## Figures and Tables

**Table 1:** Sociodemographic and clinical data of patients with and without risk of suicide.

Variable	Category	Without suicidal risk (n=238)	With suicidal risk (n=12)	*p*-value
Marital status	Divorced, separated, widowed, or single	40 (88.9%)	5 (11.1%)	**0.045**
	Married	198 (96.6%)	7 (3.4%)	
Living alone	Yes	12 (80%)	3 (20%)	**0.028**
	No	226 (96.2%)	9 (3.8%)	
Prior psychiatric treatment	Yes	19 (79.2%)	5 (20.8%)	**0.003**
	No	219 (96.9%)	7 (3.1%)	
HADS	With anxiety	21 (77.8%)	6 (22.2%)	**0.001**
	Without anxiety	217 (97.3%)	6 (2.7%)	
HADS	With depression	13 (76.5%)	4 (23.5%)	**0.005**
	Without depression	225 (96.6%)	8 (3.4%)	

**Table 2:** Sociodemographic and clinical data of patients with and without risk of suicide.

Variable	Category	Without risk of suicide (n=238)	With risk of suicide (n=12)	*p*-value
Race	White	150 (94.3%)	9 (5.7%)	0.546
	Non-white	85 (96.6%)	3 (3.4%)	
Religion	Religious	216 (95.6%)	10 (4.4%)	0.491
	Nonreligious	13 (92.9%)	1 (7.1%)	
Age bracket (y)	≤54	34 (89.5%)	4 (10.5%)	0.201
	55 to 65	117 (96.7%)	4 (3.3%)	
	≥66	87 (95.6%)	4 (4.4%)	
Education level	Primary school (complete or incomplete) and illiterate	56 (98.2%)	1 (1.8%)	0.462
	High school (complete or incomplete) and technical course	63 (95.5%)	3 (4.5%)	
	College (complete or incomplete)	119 (93.7%)	8 (6.3%)	
Place of residence	In the state of SP	225 (95.7%)	10 (4.3%)	0.156
	Outside SP	13 (86.7%)	2 (13.3%)	
Health system	Private and supplementary	177 (95.2%)	9 (4.8%)	0.999
	SUS	61 (95.3%)	3 (4.7%)	
D’Amico classification	Low-risk	102 (98.1%)	2 (1.9%)	0.130
	Intermediate-risk	136 (93.2)	10 (6.8%)	
Personal history of cancer	Yes	16 (100%)	0 (0%)	0.999
	No	222 (94.9%)	12 (5.1%)	
Family history of cancer	Yes	139 (93.9%)	9 (6.1%)	0.369
	No	99 (97.1%)	3 (2.9%)	
Actively smoking at the time of the interview	Yes	29 (96.7%)	1 (3.3%)	0.999
	No	209 (95%)	11 (5%)	
Indicated for surgical treatment	Yes	165 (96.5%)	6 (3.5%)	0.203
	No	73 (92.4%)	6 (7.6%)	
	No	200 (96.2%)	8 (3.8%)	
Family history of suicidal behavior	Yes	30 (90.9%)	3 (9.1%)	0.200
	No	208 (95.9%)	9 (4.1%)	
Drug use to aid sleeping and for anxiety or depression	Yes	38 (90.5%)	4 (9.5%)	0.123
	No	200 (96.2%)	8 (3.8%)	

**Table 3:** Probability of committing suicide.

Living alone n=15 (6%)	Prior psychiatric treatment n=24 (9.6%)	HADSa n=27 (10.8%)	N=250 (%)	Probability of suicide risk
Yes	Yes	Yes	0 (0.0%)	88.74%
Yes	No	Yes	2 (0.8%)	53.04%
Yes	Yes	No	1 (0.4%)	48.96%
No	Yes	Yes	7 (2.8%)	44.28%
No	No	Yes	18 (7.2%)	10.22%
No	Yes	No	16 (6.4%)	8.82%
Yes	No	No	12 (4.8%)	12.08%
No	No	No	194 (77.6%)	1.37%
